# Authorship diversity in Gastroenterology-related Cochrane systematic reviews: Inequities in global representation

**DOI:** 10.3389/fmed.2022.982664

**Published:** 2022-09-02

**Authors:** Arkadeep Dhali, Christopher D'Souza, Roger B. Rathna, Jyotirmoy Biswas, Gopal Krishna Dhali

**Affiliations:** ^1^Institute of Postgraduate Medical Education and Research, Kolkata, India; ^2^St. John's Medical College, Bangalore, India; ^3^College of Medicine and Sagore Dutta Hospital, Kolkata, India

**Keywords:** authorship, diversity, gender, bias, academic Gastroenterology

## Abstract

The need for promoting diversity and equitable authorship representation in academics faces increasing recognition, with some articles pointing out the lack of diversity in specific fields. Currently, there are no such articles scrutinizing the author diversity in the field of Gastroenterology. Cochrane systematic reviews are perceived worldwide to be amongst the highest quality of evidence available, thereby its conclusions often impact policy and practice globally. However, little is known about the current state of authorship diversity in Gastroenterology-related Cochrane reviews.

## Objective

This study sought to determine the sex and country diversity in authorship representation in the authorship of Cochrane systematic reviews related to Gastroenterology.

## Background

The need for promoting diversity and equitable authorship representation in academics faces increasing recognition, with some articles pointing out the lack of diversity in specific fields. Currently, there are no such articles scrutinizing the author diversity in the field of Gastroenterology. Cochrane systematic reviews are perceived worldwide to be amongst the highest quality of evidence available, thereby its conclusions often impact policy and practice globally. However, little is known about the current state of authorship diversity in Gastroenterology-related Cochrane reviews.

## Methods

### Data source

Data was collected from the Cochrane Library on 23 April 2022, using the keywords “Gastroenterology” in advanced search under the subheading “Title Abstract Keyword.” All the articles were extracted which included published reviews (current and previous versions) and withdrawn publications. Studies, where only the protocol is published to date, were excluded from the analysis. The temporal distribution of the articles ranged from 2004 to 2021. One hundred and six publications with a total of 545 authors were included in the current study. The date of publication, the title of the article, DOI (Digital Object Identifier), complete author list, and the email address for correspondence was collected.

### Author identification

The designation of the first author and corresponding author was based on the author list order and correspondence provided. Those authors who did not provide a complete name were queried on public search engines such as Google and other publications on databases (Pubmed, Research gate, Scopus, Embase, Hinari, etc.), and institutional pages. Sex (female/male) was assigned to each author based on an online search, attempting to capture at least two web pages demonstrating it (such as LinkedIn, Research gate, Loop profile, Institutional websites, Journal editorial profile etc.). For those authors whose sex couldn't be ascertained by the above methods, attempts were made to contact the corresponding author to determine their sex and that of their co-authors. The identification of sex solely based on the name or appearance was deferred until confirmation was obtained by the corresponding author. Authors whose sex could not be ascertained were excluded from sex-based analyses but were included in the country-based analysis. We treated a collaborative author group belonging to a single country, e.g., MRC Clinical Trials Unit (UK), as a single author. A second investigator independently cross-verified the extracted data.

### Statistical analysis

A Google sheet was created to collect and store the data. The collected data were presented as frequency (percentage, %) and ratios as appropriate. For a graphical representation of global diversity, we used a choropleth-style map.

## Results

**Figure 1:**
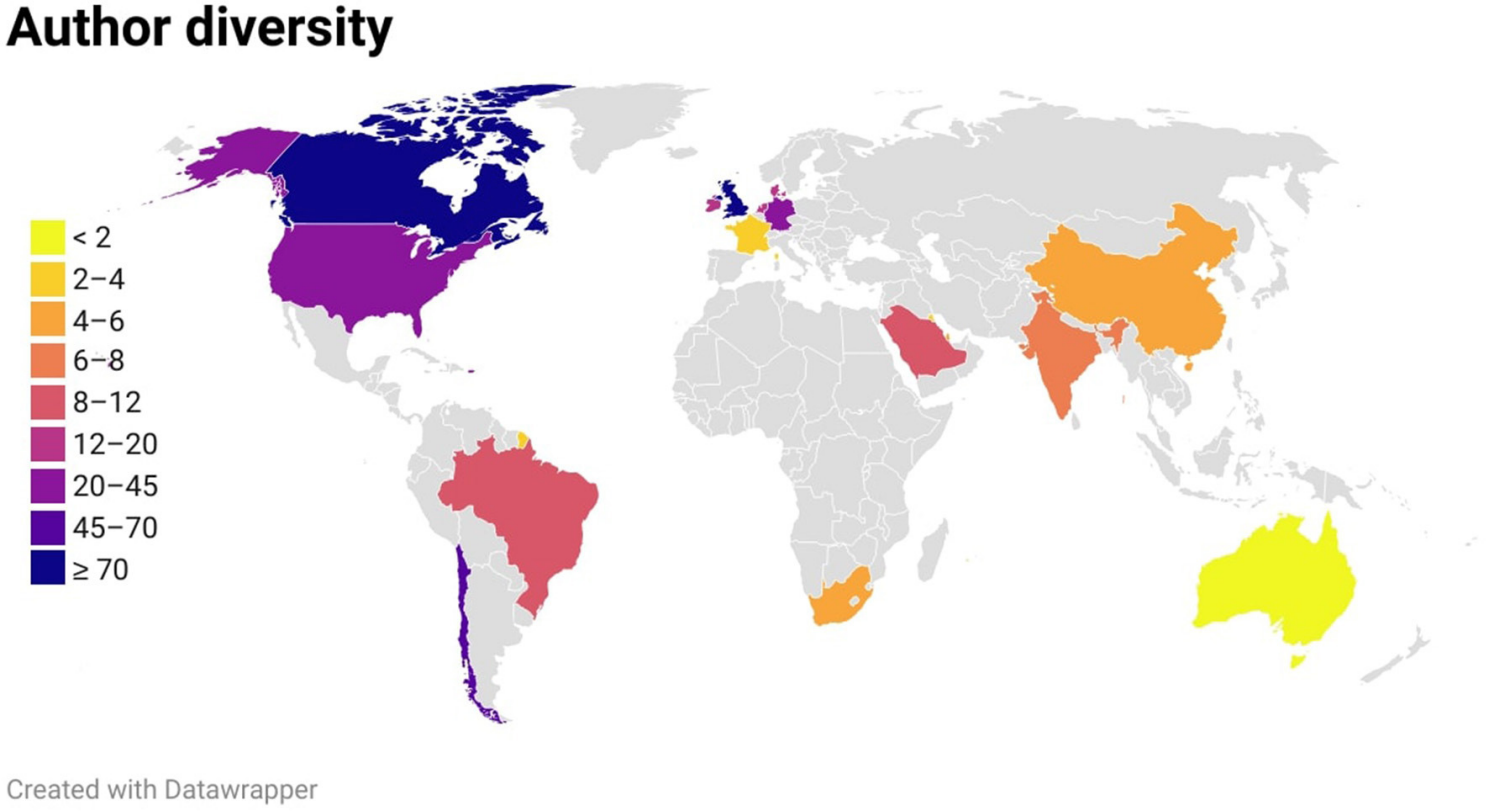
Choropleth style map showing nation-wise author contribution in Gastroenterology-related Cochrane systematic reviews.

One hundred and six publications with a total of 545 authors were included in the current study. The leading five represented nations (Figure 1) in authorship were Canada (*n* = 195, 35.9%), United Kingdom (*n* = 119, 21.9%), Chile (*n* = 69, 12.7%), Germany (*n* = 42, 7.7%), and United States of America (*n* = 30, 5.5%). First authors were mostly represented by Canada (*n* = 41, 38.6%), followed by United Kingdom (*n* = 26, 24.5%), Chile (*n* = 14, 13.2%), Germany (*n* = 6, 5.6%), and Denmark (*n* = 6, 5.6%). India is the only country among all the low and low-middle-income countries which had authorship representation and constituted 1.1% (*n* = 6) of all the authors.

**Figure 2:**
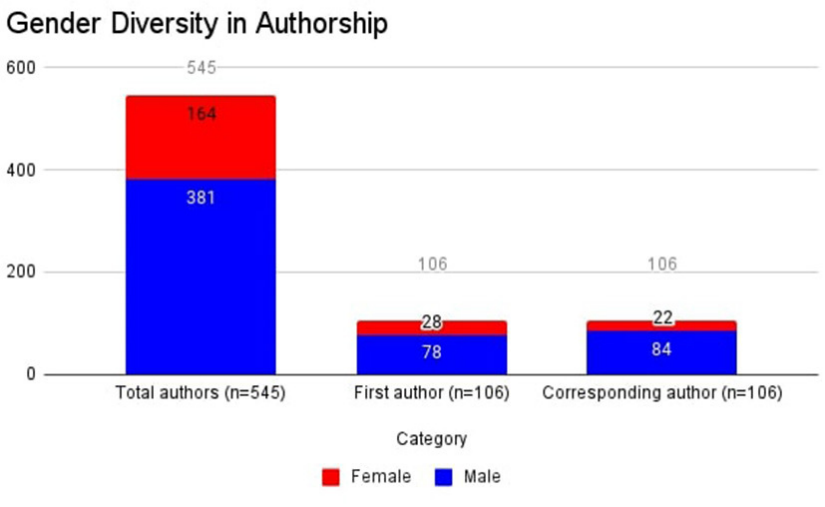
Bar chart demonstrating sex representation in authorship.

Male (*n* = 381) to female (*n* = 168) ratio in this study was 2.26:1 (Figure 2). There were 78 (73.6%) male and 28 (26.4%) female first authors (sex ratio 2.78:1). Women (*n* = 22) constituted 20.8% of all the corresponding authors (sex ratio 3.81:1). Thirty-nine (36.7%) studies didn't have any female representation in any lead author (corresponding or first author) position. Twenty (18.8%) studies didn't have any female authors at all.

The author data, apart from their research gate profile (*n* = 533, 97.7%) had other websites where their sex was obtained from. These were, LinkedIn profile (*n* = 303, 55.59%), institutional web pages (*n* = 198, 36.3%), private practice websites (*n* = 221, 40.5%), journal web pages (*n* = 42, 7.7%). The remainder of the authors were contacted individually through their correspondence (*n* = 38, 6.9%).

## Discussion

It has been well-established that there exists a disparity in sex representation in scientific research. There are far more male authors than female authors. In terms of author positions, women are underrepresented in dominant author positions (first authors and corresponding authors). The sex disparity extends further into the number of citations, where it was observed that articles with female authors in dominant positions were far less likely to be cited than the ones with male authors (1). It was also observed that male authors were more likely to be invited to write articles in journals as compared to their female colleagues (2). This difference in the representation of sex extends even in the field of medical research. It has been observed that women were underrepresented in the first authorship in high-impact journals. Although the trend has improved in the last 20 years, the difference is still evident (3). Women were also less likely to receive the opportunity of authoring an invited commentary in medical journals as compared to men with similar expertise (4). The sex disparity extends through the hierarchical levels, with women being underrepresented in leadership roles–a review found that women held a minority of editorial positions in women's health journal boards across all editorial positions (5). These differences in representations have an impact on the way women have on laying down management plans, with the number of women authors on clinical practice guidelines being significantly low (6).

The sex disparity is not limited merely to research opportunities and scientific contributions. In clinical research, there has been a long-existing practice of focusing the research question on males and extrapolating the observations to the female population. This sex bias was also very prevalent in clinical trials–with most of the drugs being tested for safety, efficacy, and adverse reactions in male populations, disregarding the differences sex can have on pharmacological effects (7). Women were excluded from early studies of drugs before 1994 (8). It was only following the guideline for the study and evaluation of sex differences in clinical trials by the US National Institute of Health (NIH) in 1994 that this practice changed (9). Sex bias in evidence-based medicine also manifests in the standardization of guidelines, where the differences in sex data have not been taken into consideration resulting in some guidelines which are not sex-specific. Knowing and acknowledging these differences is very vital since they affect standard management practices and education (7).

The jarring sex gap requires foundational changes to be implemented. However, there are also fewer women in leadership roles in journals (10). It has been observed that having women in leadership roles has positively impacted the representation of women in editorial boards as well as peer review (11, 12).

We have also found that the sex distribution among all the practicing physicians within the specialty correlates well with the patterns of author representation. Most high income countries report that the number of female physicians in gastroenterology ranged from a little <20–30%. The Association of American Medical Colleges report that only 18.9% of Gastroenterologists practicing within the country were female^1^. Similar figures have been reported across most countries, i.e., Canada (30.9%), and United Kingdom (22%) as reported by their respective medical associations^2^,^3^,^4^. However, the first authorship and corresponding authorship roles have been significantly low compared to the existing distribution of physicians. Authors from high-income countries continue to be the largest contributors to Cochrane systematic reviews in Gastroenterology, a source of one of the highest quality evidence. There is extremely poor representation of authors from low and low-middle-income countries. Sex bias is also noted with women poorly represented both as contributors as well as lead authors. A similar scenario exists in leading positions in academic Gastroenterology. In an insightful study by Sethi et al., men were listed as comprising 86% of chairs, 82% of division chiefs, 76% of program directors, and 63% of associate program directors of the Gastroenterology fellowship program in the United States of America (13). In another study by Leung and colleagues, women constituted only 7.7% of the editors-in-chief and 31 of the editorial board members of leading Gastroenterology and Hepatology journals (median impact factor 5.55) (14). It is also worth mentioning that existence of positive correlations were found between male-dominated editorial boards and male first (+0.52, *P* = 0.005) and senior authorship (+0.56, *P* = 0.002), whereas negative correlations were observed between male-dominated editorial boards and female first (−0.51, *P* = 0.006) and senior authorship (−0.56, *P* = 0.002) (14). American College of Gastroenterology has launched the #DiversityinGI social media campaign to promote inclusion in gastroenterology (15). Active capacity-building efforts are needed in several countries for advancing authorship diversity among academic gastroenterologists involved with Cochrane. This study has certain limitations. It focuses on rather limited data of only 106 publications on systematic reviews from the Cochrane Library. Further studies are required which has a more comparative nature so that, the country and sex diversity could be examined in other 'lower' strata of the evidence pyramid (randomized control trials; prospective cohort studies; retrospective cohort studies, etc.) as well as in different specialties (cardiology; rheumatology; nephrology; surgery; etc.).

## Data availability statement

The raw data supporting the conclusions of this article will be made available by the authors, without undue reservation.

## Ethics statement

Ethical review and approval was not required for the study on human participants in accordance with the local legislation and institutional requirements. Written informed consent for participation was not required for this study in accordance with the national legislation and the institutional requirements.

## Author contributions

AD and CD'S wrote the manuscript. CD'S, JB, RR, and GD performed data collection. AD conceptualized the manuscript. All authors contributed to the article and approved the submitted version.

## Conflict of interest

The authors declare that the research was conducted in the absence of any commercial or financial relationships that could be construed as a potential conflict of interest.

## Publisher's note

All claims expressed in this article are solely those of the authors and do not necessarily represent those of their affiliated organizations, or those of the publisher, the editors and the reviewers. Any product that may be evaluated in this article, or claim that may be made by its manufacturer, is not guaranteed or endorsed by the publisher.

## Author disclaimer

The abstract is presented at the International Digestive Disease Forum 2022 (IDDF2022-ABS-0056).

## Footnotes

^1^Available online at: https://www.aamc.org/data-reports/workforce/interactive-data/active-physicians-sex-and-specialty-2019# (accessed July 31, 2022).

^2^Available online at: https://www.cma.ca/sites/default/files/2019-03/2018-06-spec-sex.pdf (accessed July 31, 2022).

^3^Available online at: https://www.bsg.org.uk/wp-content/uploads/2022/03/British-Society-of-Gastroenterology-Workforce-Report-2021.pdf (accessed July 31, 2022).

^4^Available online at: https://gateway.euro.who.int/en/indicators/hfa_499-5261-number-of-physicians-medical-group-of-specialties-pp/ (accessed July 31, 2022).
